# Accounts of severe acute obstetric complications in Rural Bangladesh

**DOI:** 10.1186/1471-2393-11-76

**Published:** 2011-10-21

**Authors:** Shegufta S Sikder, Alain B Labrique, Barkat Ullah, Hasmot Ali, Mahbubur Rashid, Sucheta Mehra, Nusrat Jahan, Abu A Shamim, Keith P West, Parul Christian

**Affiliations:** 1 Department of International Health, Johns Hopkins Bloomberg School of Public Health, Baltimore, MD USA; 2 Maternal and Newborn Health Program, United Nations Fund for Population Activities Narail, Bangladesh; 3 The JiVitA Maternal and Child Health Research Project, Gaibandha, Bangladesh; 4 Partners in Population and Development, Dhaka, Bangladesh

## Abstract

**Background:**

As maternal deaths have decreased worldwide, increasing attention has been placed on the study of severe obstetric complications, such as hemorrhage, eclampsia, and obstructed labor, to identify where improvements can be made in maternal health. Though access to medical care is considered to be life-saving during obstetric emergencies, data on the factors associated with health care decision-making during obstetric emergencies are lacking. We aim to describe the health care decision-making process during severe acute obstetric complications among women and their families in rural Bangladesh.

**Methods:**

Using the pregnancy surveillance infrastructure from a large community trial in northwest rural Bangladesh, we nested a qualitative study to document barriers to timely receipt of medical care for severe obstetric complications. We conducted 40 semi-structured, in-depth interviews with women reporting severe acute obstetric complications and purposively selected for conditions representing the top five most common obstetric complications. The interviews were transcribed and coded to highlight common themes and to develop an overall conceptual model.

**Results:**

Women attributed their life-threatening experiences to societal and socioeconomic factors that led to delays in seeking timely medical care by decision makers, usually husbands or other male relatives. Despite the dominance of male relatives and husbands in the decision-making process, women who underwent induced abortions made their own decisions about their health care and relied on female relatives for advice. The study shows that non-certified providers such as village doctors and untrained birth attendants were the first-line providers for women in all categories of severe complications. Coordination of transportation and finances was often arranged through mobile phones, and referrals were likely to be provided by village doctors.

**Conclusions:**

Strategies to increase timely and appropriate care seeking for severe obstetric complications may consider targeting of non-certified providers for strengthening of referral linkages between patients and certified facility-based providers. Future research may characterize the treatments and appropriateness of emergency care provided by ubiquitous village doctors and other non-certified treatment providers in rural South Asian settings. In addition, future studies may explore the use of mobile phones in decreasing delays to certified medical care during obstetric emergencies.

## Background

Annually, approximately 9.5 million women around the world suffer from pregnancy-related complications, and over 300,000 die [1,2]. Maternal mortality in Bangladesh remains high, contributing to an estimated 3% of the global burden despite estimates of the maternal mortality ratio (MMR) having decreased from 574 in 2000 to 194 in 2010 [1]. Still, maternal deaths remain a problem in Bangladesh, with estimates of MMR as high as 782 in remote areas of the country [3]. Hemorrhage, eclampsia, prolonged or obstructed labor, puerperal sepsis and abortion-related deaths are the main causes of maternal death [2,4,5].

While MMRs provide an indication of trends in mortality rates over time, maternal deaths are difficult to study, given their rarity. Recent research has focused on severe acute obstetric complications to elucidate risk factors and potential strategies for prevention of maternal mortality and severe morbidity [6]. An obstetric complication is defined as an acute condition arising from a direct cause of maternal death, such as antepartum or postpartum hemorrhage, obstructed labor, postpartum sepsis, complications of abortion, pre-eclampsia or eclampsia, ectopic pregnancy, and ruptured uterus, or indirect causes such as anemia, malaria, and tuberculosis [7].

Similar to the major causes of maternal mortality, hemorrhage, hypertensive disorders of pregnancy, obstructed labor, complications of induced abortion, and sepsis are the leading severe obstetric complications in developing countries [8,9]. The World Health Organization (WHO) has recently standardized the definition of life-threatening, severe acute obstetric complications under the concept of near miss, which is "a woman who nearly died but survived a complication that occurred during pregnancy, childbirth or within 42 days of termination of pregnancy" [10]. Near misses are typically defined using clinical criteria that depend on the existence of laboratory facilities and intensive care monitoring [11]. Among women with severe obstetric complications, near misses are considered to have the most serious conditions, nearly resulting in maternal death.

The majority of maternal deaths and severe obstetric complications are clustered around labor and delivery [5]. Although skilled attendance at birth and facility births are promoted for reduction of maternal deaths, 85% of births in Bangladesh occur at home and only 18% of births are attended by skilled attendants [12]. (Skilled attendants are defined as board-certified doctors, nurses, midwives, paramedics, community skilled birth attendants, or government-trained providers [12]). Trained traditional birth attendants (TBAs) are reported to assist in 11% of deliveries, whereas over 70% of births are assisted by untrained traditional birth attendants, relatives, friends, or neighbors. National statistics, however, mask urban-rural and socioeconomic disparities as the likelihood of seeking medical treatment during pregnancy or childbirth increases with the mother's education, household wealth, location, and parity [4,6]. Further information on healthcare decision-making during obstetric emergencies is needed to improve understanding of care seeking for obstetric complications in rural Bangladesh.

A recent systematic review of 30 studies on near misses in developing countries revealed that the majority (80%) were conducted in hospital settings [10]. Yet hospital studies are unlikely to be representative of the general population of women giving birth, especially in settings in which most women give birth at home. In addition, many hospital-based studies ignore the well-documented delays that prevent women from accessing facility care during delivery. The literature indicates that women with severe acute obstetric complications are often referred to hospitals and clinics in late stages of emergency due to delays that include lack of recognition of severity of maternal complications [13-15], lack of preparedness for an emergency [16], women's lack of decision-making power regarding prenatal care and place of delivery [15], and perception of inadequate or poor quality hospital care [17]. Barriers that prevent women from accessing hospitals are multiple and compounded in Bangladesh, where the majority of births and maternal complications occur in the home in the absence of skilled attendants [4]. Contextual data on decision making and care seeking during severe acute obstetric complications in community settings of South Asia is still lacking. We conducted a qualitative study of women reporting severe acute obstetric complications in order to identify a broad spectrum of underlying social, familial, economic, knowledge, and empowerment factors in the healthcare seeking process and the major barriers perceived by women in accessing appropriate care. Our goal was to elicit the perspectives of women who reported having life-threatening severe obstetric complications in order to highlight barriers and facilitating factors that allowed women to receive life-saving care during obstetric emergencies in a rural, representative, community setting. This study aims to provide a community-based perspective to the literature on severe obstetric complications.

## Methods

### JiVitA-3 Study Context and Procedures

The present study was conducted from October to November 2009 in Gaibandha District in rural, northwest Bangladesh. The levels of infrastructure, maternal and child nutrition, socioeconomic and health status, and patterns of health care use in the area are typical of lower socioeconomic rural communities in the country [18]. The MMR and neonatal mortality rate in the study area is reported as 289 and 60, respectively, while literacy rates range from 31% among males to 17% among females [12,18]. The study was nested within an ongoing, cluster-randomized trial called JiVitA-3 that is assessing the effect of daily, multiple micronutrient supplementation during pregnancy in reducing infant mortality. JiVitA-3 began in 2007 in a cohort of ~130,000 married women of reproductive age (aged 14 to 45 years) from whom approximately 43,000 pregnancies will be recruited over 3.5 years of enrollment.

The parent trial, through collection of prospective follow-up data on women with severe obstetric complications, provided a unique opportunity for nesting this research. Pregnant women are identified using a urine-based test and enrolled for supplementation after obtaining informed consent. Pregnancy supplementation and outcomes are monitored on a weekly basis. One month after a pregnancy outcome, trained female interviewers gather a history of postpartum morbidity symptoms, at which time women are asked if at any time they felt that they had nearly died during pregnancy or in the 30 days following the end of pregnancy. Women reporting such a crisis are asked a series of structured questions on the morbidities they experienced during this event, the type of health care they sought, and the type of transportation they used to reach health facilities. In an open-ended narrative section, women are also asked to describe the event in detail, in their own words. In this study, we define women with severe obstetric complications as those who said they felt that they nearly died during pregnancy, delivery, or 30 days postpartum and who reported seeking care for this complication. We used care seeking as a proxy for severity in order to capture severe obstetric complications. In settings in which home-based births are the norm, women's report of care seeking for a life-threatening complication has been considered a proxy for illness severity [19-22]. Since our study is based on self-report and not on clinically confirmed events, we refer to life-threatening pregnancy-related complications as severe acute obstetric complications rather than near misses.

In this study area, approximately 90% of births occur at home. Treatment providers range from non-certified providers such as traditional healers, village doctors, shamans, traditional birth attendants, and homeopathic doctors to certified providers who are authorized to provide emergency obstetric care, such as doctors, nurses, midwives, or government-trained providers such as family welfare visitors. Traditional healers and shamans, generally visited for conditions that are perceived to be caused by evil spirits, provide blessings against these spirits [23]. Most traditional healers do not have their own shops, but rather are known in the community and are called to visit homes for treatment. The ubiquitous village doctors include a wide range of providers, some of which have three to six months of training on allopathic treatment, while others work in medicine shops but otherwise lack training. Village doctors provide primary allopathic treatment through saline injections, capsules, tablets, antibiotics, or ointments [23]. In this community, some homeopathic providers have an academic degree on homeopathy, while others who learn by apprenticeship [23].

Certified providers, including doctors, nurses, midwives, and government-trained providers, often work in government clinics or hospitals such as medical college hospitals (located in larger districts), district hospitals and maternal and child welfare centers, *upazila *(sub-district) health complexes, and union health centers (located at the lowest administrative level) [24]. In government hospitals, maternity care is provided for free, though studies estimate that hidden costs are associated with medicines and other supplies [25]. The 2010 Bangladesh Morbidity and Mortality Survey reports that all medical college hospitals, district hospitals, and selected *upazila *health complexes and maternal and child welfare centers provide comprehensive emergency obstetric care (a package of medical interventions which includes C-section and blood transfusions) [26]. Yet facility assessments of public hospitals in Sylhet (a district in northeastern Bangladesh) demonstrate that actual availability of services is lower than reported availability [24]. While much of the growth in institutional delivery in Bangladesh is attributed to private facilities, these facilities do not routinely report to the government on the types of services that are provided [24]. Maternity care at private facilities is provided at cost, which varies depending on the clinic and setting.

**Figure 1:**
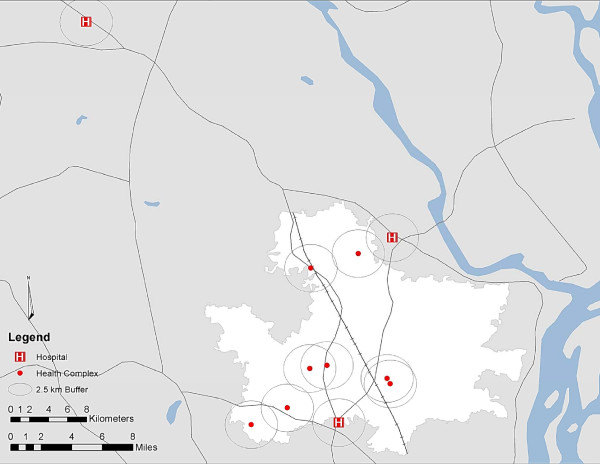
**Pathway to receipt of emergency medical care during severe obstetric complications**. Figure 1 illustrates the government clinics that are commonly visited by women enrolled in the JiVitA-3 study. The study area is shown in white, with main roads shown in gray. The red dots denote the maternal and child welfare center and sub-district hospitals, which are reported to provide basic emergency obstetric care. The H's near the boundaries of study area indicate the two district hospitals, while the H to the upper left quadrant indicates the nearest medical college hospital, located 48 kilometers from the center of the study area. The distance to the medical college hospital, one of the only facilities in this area reported to provide comprehensive emergency obstetric care, illustrates the geographic barriers that residents have to overcome to seek referral services.

The study area contains eight government health complexes, including one maternal and child welfare center and seven sub-district hospitals (shown in Figure 1). There are two district hospitals located close to the study area. The nearest medical college hospital that is visited by study participants is located 48 kilometers from the center of the study area. Comprehensive emergency obstetric care is provided by district hospitals and the medical college hospital, while the sub-district hospitals and the MCWC are reported to provide basic emergency obstetric care.

### Selection of Participants and Interviews

For this qualitative study, participants were identified from the pool of women who reported that they felt that they nearly died due to a severe obstetric complication. Selection criteria included: 1) reporting a severe obstetric complication in the last 12 months (to reduce recall bias) and 2) reporting having sought care for complications. Care seeking was defined as having called a health provider to the home or having visited a clinic or hospital. Women were classified into broad "morbidity groups" designed to reflect major causes of maternal mortality (Table 1). Classifications were based on WHO recommendations for applying International Classification of Disease codes to settings lacking vital registration systems and largely dependent on verbal autopsies [27]. From a pool of 205 women identified as reporting severe obstetric complications, 40 women who represented the top five most common obstetric complications were purposively selected. Of the 40 women interviewed, nine reported morbidity symptoms consistent with hemorrhage, eight with obstructed labor, seven with eclampsia, seven with sepsis, and nine with induced abortion. Women who reported symptoms consistent with more than one morbidity group were excluded to reduce the chance of misclassification.

**Table 1:** Case definitions for leading severe obstetric complications

	Study Definition	**WHO definition in verbal autopsy setting **[27]
Hemorrhage	Profuse bleeding AND severe pallor	Pregnancy > 8 months, Severe vaginal bleeding^a^

Puerperal Sepsis	High fever in the 7 days after delivery	High fever AND having Vaginal delivery
		> 1 day ago OR having Caesarean section
		> 1 day ago

Eclampsia	Convulsions, excluding epilepsy [whole body swelling OR severe dizziness] AND NO high fever	Being pregnant > 5 months, delivery < 6 weeks ago, swollen upper extremity and/or face, convulsions, visual disturbances, hypertension, first birth, gastric pain, headache, and no fever

Obstructed Labor	Baby stuck at delivery AND length of labor >24 hours	Physical obstruction + >24H Labor

Induced Abortion	"Something done to end pregnancy" AND use of invasive procedure [e.g. insertion of object OR Menstrual Regulation OR D&C]	Termination of pregnancy or therapeutic abortion

^a ^The timing of the hemorrhage is used to classify the event as antepartum or postpartum. Severe vaginal bleeding before delivery is considered to be antepartum hemorrhage, while severe vaginal bleeding and delivery less than three days ago is considered as postpartum hemorrhage.

Interviewers were conducted by the first author (SSS) and two assistants (all were Bangladeshi females). The primary interviewer was fluent in Bangla (the local language), trained in qualitative methods, and experienced in conducting qualitative interviews with rural Bangladeshi women. The assistants, both quality control interviewers with the parent study, had nine years of experience in conducting interviews on maternal and neonatal morbidities and were familiar with the local dialect. They received one month of training on qualitative methods. The first five interviews were observed by a senior female Bangladeshi supervisor with a master's degree in anthropology (NJ) to provide feedback to the team. She also completed six random spot checks as part of ongoing quality control.

### Data Collection and Procedures

The semi-structured interview guides were developed in consultation with the project anthropologist (NJ) to ensure cultural relevancy. The in-depth interview guide began with an open-ended section that asked, "Tell me, in your own words, about what happened when you were seriously ill during your pregnancy." This question was followed by 22 sets of items on a) detailed description of the severe acute complications, b) healthcare decision making during the obstetric emergency, c) treatment-seeking behavior during pregnancy, d) and lessons learned from the event. Contextual information such as the woman's demeanor and emotion was also recorded. Probes such as "What do you mean when you say "..."?", "How did ...this happen?", and "Can you give me an example of ...?" were used for clarification and enrichment of descriptions.

JiVitA-3 field supervisors visited each selected woman to explain the purpose of the study, and to schedule a visit by the interviewers. Next, the in-depth interviewers visited the woman and explained the purpose of the study and obtained informed consent. Interviews were conducted in the local language, with most interviews lasting for about an hour. With consent, the interviews were also audio-recorded to facilitate review of field notes. Interviews were conducted with only the woman and interview team present.

### Data analysis

Within 24 hours following each interview, the interviews were translated by SSS into English and used to expand the field notes. Detailed field notes were analyzed in Atlas.ti qualitative analysis software. Each interview was read line by line to code keywords and to identify recurring themes that emerged across interviews about healthcare decision makers, the woman's perception of barriers to receiving timely care, and her family's perceptions of the emergency. Coding was reviewed and enhanced by a second reviewer (NJ). Identified themes were further explored using code families and network maps to build a conceptual model of the most commonly shared perceptions of barriers or facilitating factors to receiving medical care for severe obstetric complications.

The study was reviewed and approved by the Johns Hopkins Bloomberg School of Public Health Institutional Review Board and the Bangladesh Medical Research Council.

## Results

### Common Characteristics

The average woman was 23 years old at the time of the crisis event and had been pregnant twice. About a third of the women had no schooling and one third lived in households that owned one mobile phone. All women were members of at least one microcredit program, which typically provided funds for small businesses, while some provided support for education. The interviewed women, on average, were younger and had lower parity compared to the overall study population (data not shown). Half reported having had at least one antenatal care (ANC) visit during pregnancy. Reasons given for not having ANC checkups included lack of support and/or money from their husbands and the perception that these visits were unnecessary if the women had had previous uncomplicated pregnancies.

Interviewed women were married at an average age of 13.5 years. Parents or relatives, rather than the women themselves, determined these unions. One woman, married at the age of 13, explained that she was not even informed of her marriage until she arrived at what she realized was her marriage ceremony. Respondents who wanted to remain in school were not supported by their families once they were married. A respondent married at the age of 12 said, "My in-laws said I would be able to study after I got married, but I became busy with cooking for everyone and they always kept me busy with housework." Shortly after marriage, most women said they were pressured by their in-laws to have children. A 27-year-old woman with two children, the only one interviewed to have passed her higher secondary exam, had worked as a schoolteacher before marriage. "I was forced to quit by my husband and mother-in-law since they did not approve of a career or study for me," she said. "I have to raise my daughter, but when she is older I will try to go back to work. I miss having a career."

Cultural practices like polygamy, although rare, also limited women's decision-making power. Four women (10%) reported polygamous marriages, and three of them said their husbands had married additional wives without informing them. They complained that their husbands did not pay enough attention to their family's needs. "I have to marry off my eldest daughter, who is 16," said a 32-year-old woman who had an induced abortion. "I wanted her to finish metric [tenth-grade certification] first, but I did not have money to pay for books. My husband does not provide much money; he is not even able to feed us properly. I have no men in the family to look after my health needs."

**Figure 2:**
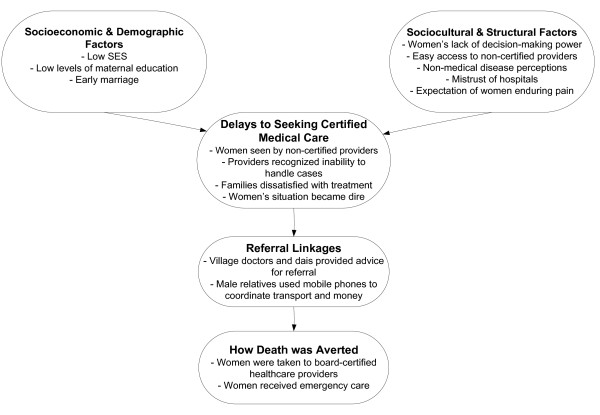
**Pathway to receipt of emergency medical care during severe obstetric complications**. This overall conceptual model depicts the common factors reported to contribute to delays in seeking certified care as well as facilitating factors that allowed women to eventually receive life-saving care. Barriers are organized into socioeconomic and demographic factors and sociocultural and structural factors. Advice from non-certified providers and enhanced coordination through use of mobile phones enabled women to receive emergency care from certified providers.

The overall conceptual model depicts the factors that women discussed as contributing to their severe obstetric complications and the ways in which they were able to avoid death (Figure 2). Socioeconomic factors, such as low maternal education and early marriage, may have contributed to delays in seeking medical care from skilled providers.

### Context of Labor and Delivery

Of the 40 women interviewed, 29 women experienced live births and 11 experienced stillbirths. Most women described labor pain as *nabi gora theke batha *(pain arising from the umbilical cord) that was much greater than abdominal pain they had experienced during pregnancy. They generally informed only a female member of their family, such as their mother or aunt, who usually advised them to keep silent and endure their pain. Maintaining silence was seen as a measure of mental and physical composure, while women who were verbally expressive of their pain were considered to be undisciplined. A 24-year-old woman who reported postpartum hemorrhage explained, "I told my aunt that I was having labor pain, and she told me not to tell anyone." Echoing the sentiments of many women, a 30-year-old woman who reported symptoms consistent with eclampsia said, "I decided to try [giving birth] by myself at home because it [childbirth] is a matter of *sharam *[shame]." Some women indicated that they kept silent so that they would have an easier delivery. A 30-year-old woman who had postpartum hemorrhage said, "I didn't tell anyone about my labor pain because it is a belief that the more people you tell about your delivery, the more difficulty you will have. There is no point in going through excessive pain."

Of women who had live births, all of them delivered at home and said that they preferred to give birth at home in order to maintain privacy. Women were encouraged by their parents and husbands to stay at home during the birth to avoid the gossip they feared they would endure if they left their homes for health care. In some cases, women explained that their husbands delayed the decision to seek timely medical care. A 32-year-old woman who had obstructed labor said, "My husband said we still had time and we should wait to call a doctor. He didn't want me to seek treatment or take medicines because if you take medicines, people gossip about you and say that you have no decency." Women explained that their neighbors believed pregnancy was a matter of shame and that women should act modestly by avoiding excessive travel in public. Women feared traveling through their communities for ANC visits or to obtain medicines because they worried their neighbors may spread rumors that they lacked moral character. An 18-year-old respondent reported that her mother insisted that she give birth in a clinic to avoid "all of the village people who enter the home during birth." This perspective, however, was rare.

Only four of the 40 women reported having medically trained assistants present during delivery. During labor, 18 women called female relatives or neighbors, who typically called one or two *dhathris *(the local term for untrained traditional birth attendants) for assistance. Fourteen women were attended by *dhathris*. Most women had decided ahead of time that they would call these *dhathris *based on their rapport with the family, similar social status, and reputation for assisting women at home during deliveries. Some women chose to call *dhathris *who had assisted them or their relatives (typically aunts or cousins) during previous deliveries. *Dhathris *were reported to perform tasks such as holding the woman's waist during delivery, encouraging women to bear down, pushing on the woman's stomach, inserting fingers into the vaginal canal to check the progress of labor, cleaning and washing the baby, and pulling out the placenta. The *dhathris*' tasks were not limited to assistance during delivery. A 28-year-old woman with two children said that her *dhathri *also helped with housework before and after she gave birth, while another woman said that her *dhathri *cleaned her up after delivery. This latter task was significant, as women are often considered to be "polluted" or unclean after childbirth.

### Care Seeking during Severe Obstetric Complications

**Figure 3:**
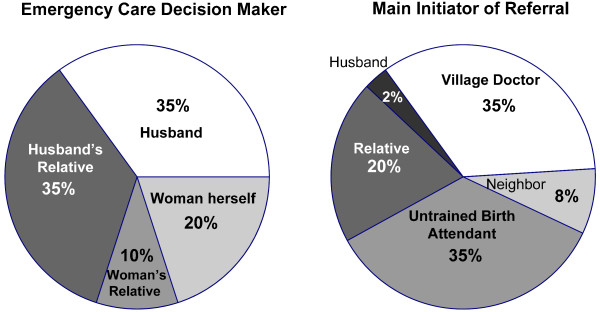
**Chief decision makers and initiators of referral during obstetric complications, including post-abortion complications**. These charts illustrate the most important actors during the health care decision-making process. The chart on the left shows the primary decision-maker during the obstetric crisis as reported by the interviewed women. The chart on the right illustrates the main person who coordinated referral to certified providers once the woman's situation became dire.

About half of the interviewed women said that they waited until they could no longer endure their pain to inform their families of the severe obstetric complication. Once they informed their families, their relatives played a central role in deciding when and where to seek care during pregnancy crises (Figure 3). More than one-third of women identified their husbands as the main healthcare decision maker, while 35% listed other male relatives, such as fathers, fathers-in-law, and uncles. Even if the husband was absent during the crisis event, some families sought the husband's permission by mobile phone before seeking care. Male relatives, including fathers, brothers, and in-laws, played an important decision-making role as women often reported going to their father's home, especially for a first birth or if the husband was not present. While female family members such as mothers-in-law, mothers, and sisters-in-law were important during the process of labor, women explained that the ultimate care-seeking decisions during their crises were made by their male relatives.

When women experienced maternal complications, 93% were first seen by non-certified healthcare providers, or providers who lacked formal medical certification or training, including village doctors (53%), *kobirajs *(traditional healers) or shamans (21%), and homeopathic doctors (19%). Women listed proximity of non-certified providers, flexibility in payment schemes, and familiarity with these providers as reasons that their family sought care from these sources. Most often, male family members called village doctors to the home since they did not require full payment upfront. In some instances, the family did not want to seek medical care as the illnesses were perceived to be non-medical in origin. A 24-year-old woman who reported having obstructed labor explained, "I did not want to have a Caesar [C-section]. I had been possessed by a *doshi *[evil spirit] when I was two months pregnant, and the *doshi *traveled in my body and gave me this problem. I needed treatment from a *kobiraj *[traditional healer] for this *doshi*, not a Caesar."

The majority of women felt that their husbands and/or other male family members had delayed the seeking of medical treatment from a certified provider. A 16-year-old respondent who had obstructed labor described her frustration, also mentioned by several other respondents, that her opinions were not taken seriously: "I wanted to call the doctor. I was so sad that my husband said we should wait longer. I was trying so hard. I didn't want to go through so much pain just so we wouldn't have to spend money." A 17-year-old respondent explained, "I knew that my condition was very serious, and everyone kept on telling me to try having the baby at home. I was trying, and I knew I couldn't try anymore, but the others didn't understand how serious it was."

Families and women usually hesitated to go to the hospital for fear of the hospital environment. Often, neighbors or relatives had told them that the government health facilities were crowded and did not maintain appropriate levels of privacy. In addition, families feared that the woman would be "torn" if a C-section was required. A 16-year-old woman who reported having eclampsia said, "No one ever wants to have a Caesar [C-section]; everyone knows it is best to have your child at home. However, we had no choice." Others feared criticism from their neighbors. A 32-year-old woman who also reported eclampsia said, "I prayed that I wouldn't have to go to the clinic. People say you are weak if you seek medical care." Other women worried about the inability to perform all of their duties if they had to recover from C-sections.

### Narrowly Avoiding Death

Once women were seen by non-certified healthcare providers, the providers typically said that they could not handle the emergency situation and advised the family to seek medical treatment at a hospital or clinic. In many cases, women described the village doctors as helping to arrange transportation. A 19-year-old woman who reported symptoms consistent with eclampsia explained, "The village doctor said that he could not handle my condition, and that I should be taken to a hospital or I would die. He gave my husband the mobile number of an ambulance." Sometimes the *dhathris *and the village doctors gave conflicting advice. A 22-year-old woman who reported having sepsis said, "The doctor told us to go to the hospital. However, the *dhathri *said that I would die on the way if I tried to leave, so we stayed at home and my uncle called another village doctor." In other cases, the families themselves realized that the treatment provided by the non-certified provider was insufficient to deal with the severity of the complications.

Village doctors and *dhathris *were the most likely to advise the husband and other family members to seek care from board-certified medical providers at clinics or health facilities (Figure 3). The husband or male relative used a mobile phone in 75% of these narratives to make arrangements for transportation or money. Most women (55%) were transported to a hospital or clinic by "rickshaw-van," a bicycle-powered flatbed cart. A 22-year-old woman who had postpartum hemorrhage recounted this rough journey: "Every time we went over a bump my bleeding would get worse." Some women were taken to three different treatment providers by rickshaw van. Women who traveled long distances often used multiple forms of transportation, such as ambulances, buses, and rickshaw-vans.

More than two-thirds of women interviewed said they had not planned for emergency medical expenses ahead of time. Although all women acknowledged the importance of birth preparedness, two-thirds of women said they were not capable of keeping money aside for their birth since they often faced difficulty in meeting their short-term daily or weekly expenses. A 19-year-old woman who had hemorrhage explained, "I had tried to keep some money ahead of time, but we didn't have anything to eat and my husband used the money to buy food." One-third of women interviewed, however, did set some money aside for birth expenses. However, their medical expenses often exceeded their expectations. To pay for medical care during their obstetric complications, seventy percent of women borrowed money from relatives, and 18% from local money lenders. These money lenders often charged very high interest rates that families repaid by selling their lands or possessions such as cows or goats.

Once families had exhausted their options of non-certified treatment providers and/or women's conditions became dire, 70% of women reported being taken to board-certified doctors, nurses, or midwives at clinics or hospitals. Women explained that they were ultimately taken to certified providers because their situation had become desperate and their families realized that the non-certified providers would be unable to handle the complications. Following care from non-certified providers, families usually went first to government facilities since treatment costs were lower than in private facilities. However, the general impression of government facilities was that they were crowded or unable to handle complicated cases. Women visiting government clinics typically report being referred to other private facilities. A 32-year-old woman who had an induced abortion described the shortage of qualified doctors in government health facilities: "There is no certainty about our government doctors here." Many families went to more expensive private facilities after government hospitals turned them away due to insufficient bed space. Once admitted to these facilities, women reported having emergency medical care including C-sections and/or transfusions.

When asked to describe the ways in which they felt their lives had been saved, the majority of women credited the divine grace of "*Allah" *for saving them from death. Seventy percent of women also attributed emergency care from a board-certified health provider as having saved their lives. Women cited the use of mobile phones by their male relatives to coordinate logistics and finances as having helped them reach emergency care in time to save their lives (Figure 2).

When asked what they would do if faced with future obstetric emergencies, 70% of women said that they would like to seek medical care from a board-certified doctor. Some women were pleased with hospital care they received from private clinics, which they had previously feared. A 17-year-old respondent with reported hemorrhage explained, "I should have gone to the [private] clinic earlier. If I had, I wouldn't have needed so much care later." Yet many women recognized that the final decision on where they sought care rested with their husbands, who most women felt would still want them to stay at home for birth. A 20-year-old woman who had obstructed labor explained, "My husband will force me to stay in our home. No matter if I live or die, I must stay at home."

### Induced Abortions

We discuss induced abortions separately since they differ from the other complications. Though "menstrual regulation," the vacuum extraction of intrauterine content to stimulate menstruation and terminate pregnancy [28], is a legal procedure in Bangladesh, most women were secretive about the fact that they had an induced abortion. MR services are reportedly available at all major government hospitals and health facilities and are legal for pregnancies up to 8 weeks. Providers authorized to perform MR include physicians, trained nurses, or family welfare visitors [29]. Yet studies indicate that untrained TBAs also perform MRs, sometimes in their own home [29,30]. Though MR is to be provided for free at government institutions, research shows that women often pay fees of at least 100 Taka ($1.4). The cost of MR is reported to range from 100 to 4000 Taka ($1.4 to $56) based on the duration of pregnancy and the location of the service provided. Costs are greater for procedures performed after the first month of pregnancy as well as those performed in private facilities rather than government institutions [29].

Nine women described nearly dying following an induced abortion, three of whom had not informed anyone else about the abortion. Reasons women gave for terminating pregnancies included poverty, existing illness, and having young children (Table 2). Most women described using two to three different methods for pregnancy termination, usually starting with a crude method such as the insertion of uterotonic tree roots into the vaginal canal and ending with menstrual regulation. Women used a particular method of pregnancy termination because they had either heard about the method from other women (female relatives or female neighbors) or because other methods were too costly.

**Table 2:** Characteristics of severe acute obstetric complications due to induced abortion

	Description	Exemplary Quotes
Reasons women wanted to terminate their pregnancies	• Existing illness	1. "I had jaundice and chest pain for many months before I became pregnant. I was very sick, and the doctor said that keeping the baby would be bad for my health." 2. "I have had many illnesses, and I do not have the strength to bear another child."
	• Lack of money	1. "We often do not have enough food or money in the home, and I do not know how we would pay for another child." 2. "My husband is not able to feed me or my daughters. How will we keep another child?"
	• Enough children or young child	1. "We already have 2 children, and I want to raise my youngest son properly." 2. "I already have a little girl, and I want to raise her well." 3. "I already have 3 girls, and I feared having another girl."

Reasons women used a particular method of pregnancy termination	• Other women told her to use this method	1. "My sister told me to use tablets for pregnancy termination." 2. "Some women who live near me had told me before that they went to a nearby bazaar to have an MR. Everyone goes, so I decided to go there to end my pregnancy."
	• Other methods were too expensive	1. "After much thought, I decided to use tree roots to end my pregnancy. I did not have enough money to buy pills to end the pregnancy." 2. "I did not seek a doctor's advice for ending my pregnancy because the treatment was too expensive. Instead, I went to a kobiraj, who gave me an amulet and herbal medicines."
	• Other methods proved ineffective	1. "Even though I used homeopathic medicines and herbal medicines to end my pregnancy, I knew the pregnancy did not end since I did not lose any blood. I then went to the community clinic to have an MR." 2. "I had to go to the hospital because I had very heavy bleeding the day after I inserted tree roots to end my pregnancy. The doctor said I was still pregnant, and that I would stay sick until I ended the pregnancy. I had an MR that day."

Reasons family members were angry about pregnancy termination	•Woman had used an unsafe method	1. "My aunts had agreed that I should terminate my pregnancy. But they got mad that I had used tree roots and yelled at me." 2. "My parents had told me to end the pregnancy, but they yelled at me because I had used homeopathic medicines."
	•Woman had not informed husband of her pregnancy or abortion	1. "My husband was working far away from home when I became pregnant. I terminated the pregnancy without telling him. He became very angry when he realized what I did." 2. "My husband wanted me to keep the baby in case we had a boy. But he worked far away from home; after he left I had an MR."

MR = Menstrual regulation

Induced abortion is the only category in which women commonly reported themselves as the main health care decision maker. Most women in this group had not informed their husbands that they were pregnant. A 31-year-old woman said, "I made the decision to terminate the pregnancy alone, after a lot of thought." Citing her family's poverty, a 23-year-old woman said, "I decided to terminate the pregnancy because I already have two children and I have to raise them properly." Further exemplary quotes are shown in Table 2.

Female contacts proved important sources of information and advice for pregnancy termination. One woman said her sister advised her to use pills to end her pregnancy, while another said her mother gave her the same advice. A 36-year-old woman said, "Everyone inserts *gacher dal *[tree branch] when they want to terminate pregnancy." Five women took what they referred to as *khoiri *tablets (brown colored tablets) on an empty stomach as the first method of terminating their pregnancies. These pills may refer to the placebo pills in oral contraception packets, which, in Bangladesh, typically contain iron [31]. Two women said they did not have money to buy tablets, and thus inserted uterotonic tree roots in the vaginal canal, while two other women took homeopathic medicines.

Following the use of crude methods, all women experienced complications, such as excessive bleeding, abdominal pain, headache, and chest pain, for which they sought care. Seven women who went to a hospital for reported hemorrhage said that the doctor informed them that their condition was life-threatening and necessitated menstrual regulation to remove the fetus. The remaining two women had called village doctors to their home for saline injections and antibiotics. While all women said they were aware of the MR procedure, five women said they initially sought other methods of pregnancy termination because they could not afford menstrual regulation.

In two-thirds of cases, the husbands worked away from home and did not learn about the pregnancy termination until after the pregnancy had been terminated. According to the respondents, their husbands frequently expressed anger that they had not been informed. Some relatives expressed anger that the woman had used unsafe abortion methods. All women reported current use of a modern family planning method (such as a contraceptive pill, intrauterine device, injectable, or male condom) to prevent future pregnancy. When asked what they would do if they accidentally became pregnant in the future, two-thirds of women said they would have menstrual regulation, while one-third responded that they would not have another abortion as they had previously experienced so many complications.

## Discussion

In our data, the majority of women appeared to understand the severity of their complications and expressed the desire to seek prompt medical care during future obstetric emergencies. Although women recognized the severity of their complications, this recognition did not translate to prompt seeking of medical care since health care decisions were made by others. Women cited their inability to convince family members or to obtain permission from their husbands as major obstacles to seeking timely medical care. Though families in these cases were able to access emergency care in time to save women's lives, the women complained of the tremendous pain they had to endure before their male relatives decided it was necessary to seek medical care. Interventions to improve timely seeking of medical care for obstetric complications may need to more effectively and appropriately target husbands and family members with messaging on care seeking. Previous studies describe early age at marriage and low maternal education as factors associated with lack of decision-making power among women in rural South Asia [17,32]. Significant improvements in care seeking may require larger societal improvements in the status of women to increase the value that is placed on women's health.

When families did decide to seek care, all women first visited non-certified treatment providers, citing established relationships, lower cost of treatment compared to certified providers, and close proximity. Such care-seeking patterns are consistent with findings from previous studies in Bangladesh. A 2001 study conducted in all six major regions of Bangladesh revealed that 89% of the 18,117 women who sought care for one or more life-threatening complications during pregnancy first sought healthcare treatment from providers who lacked formal training or certification, such as herbal or homeopathic treatment providers [33]. In our study, non-certified providers, usually village doctors or untrained birth attendants, sometimes helped refer women to higher-level providers with the use of mobile phones when they were unable to treat serious complications.

While non-certified providers linked women to life-saving care, other studies have found that inappropriate care by these first responders may endanger the lives of women in crisis situations. In selected referral hospitals in Jakarta, half of maternal deaths were related to inappropriate care by first providers (mainly midwives and TBAs) [34]. The International Center for Diarrheal Disease Research, Bangladesh reports that harmful and inappropriate use of drugs is widely prevalent among village doctors [35]. Further exploration of the types of treatment women receive from these first responders is needed.

Induced abortions were the sole morbidity category for which women made their own healthcare decisions. Early pregnancy ascertainment may play a role in allowing women to make their own decisions regarding their pregnancy, sometimes without informing their husbands or families. Women who underwent induced abortions seemed to circumvent the male-dominated, decision-making hierarchy. While menstrual regulation has been attributed to a marked decline in abortion-related deaths over the last few decades [30], induced abortions conducted by untrained TBAs remain a major cause of morbidity in developing countries [32]. Women in our study reported seeking crude methods of pregnancy termination due to their inability to pay for medical abortions. Access to safe abortion methods needs to be emphasized for women who wish to terminate their pregnancies. Post-abortion care and follow-up referral pathways are also needed to support women after pregnancy termination.

## Limitations

Although a major strength of interviewing women who survived severe obstetric complications is the ability to gain firsthand information, some women acknowledged that they were too sick during their crisis to remember all the details of the event. Interviews with husbands or relatives may have provided further information on the decision-making process and details of the care-seeking process during severe obstetric complications [17]. In addition, validation of self-reported complications may have allowed for comparison with hospital-based data on complications. We were unable to perform this validation due to poor record-keeping in facilities and issues of patient confidentiality. Thus, the care seeking patterns for complications may differ between women included in this analysis and women who are medically classified as near misses. However, the morbidity classifications used for this analysis were restricted to WHO recommendations for complication categories in verbal autopsy settings.

Although this study interviewed women who experienced complications during pregnancy, delivery, or 30 days postpartum rather than up to 42 days postpartum, we expect to have captured most severe obstetric complications since they are clustered around labor and delivery [36]. Our study minimized recall bias by asking women about severe obstetric complications within one month following pregnancy. By presenting data from a representative, rural sample, this study provides a community-based perspective to a field that has been largely dominated by hospital-based literature.

## Conclusions

While male decision-makers typically dominate the health-care decision-making process during obstetric crises, female relatives are vital sources of advice and support when women make their own decisions about sensitive procedures such as induced abortions. Established relationships with community members, flexible payment options, and geographic proximity suggest that non-certified providers will remain first-line providers for women during obstetric emergencies in these settings. Strategies to prevent maternal mortality may focus on ensuring that these first-line providers do not perform harmful procedures, provide effective referral, and are able to provide some level of appropriate emergency services. Identification of optimal strategies for improving treatment and referral among non-certified providers requires further research attention. While Bangladesh continues to move towards increasing skilled attendance at birth and facility-based births, improving treatment and referral by non-certified providers may provide a pragmatic interim strategy for the vast majority of women who continue to give birth at home.

The use of mobile phones holds potential for decreasing delays to receiving hospital care by increasing access to information and facilitating coordination of finances and logistics for emergency medical care. Reducing maternal deaths in rural Bangladesh will require the cooperation of the health providers who are first responders for women facing crises and the families who make the healthcare decisions for them.

## Competing interests

The authors declare that they have no competing interests.

## Authors' contributions

SSS provided data acquisition, data analysis and interpretation, and primary drafting and editing of the article. ABL provided primary conception and design. SSS and ABL were responsible for analytic design, revision of the article, and final approval. PC, SM, MR, KPW, and AAS contributed to conception and design of the study, critical revisions of the article, and final approval. BU and HA assisted with data acquisition, revising the article, and final approval. NJ assisted with conception and design, interpretation of data, and critical revision of the article.

All authors read and approved the final manuscript.

## Pre-publication history

The pre-publication history for this paper can be accessed here:

http://www.biomedcentral.com/1471-2393/11/76/prepub
